# Availability of Key Essential Medicines in Public Health Facilities of South Indian Union Territory: One of the Crucial Components of Universal Health Coverage

**DOI:** 10.7759/cureus.19419

**Published:** 2021-11-09

**Authors:** Dinesh K Meena, Mathaiyan Jayanthi, Kesavan Ramasamy, Mahalakshmy T

**Affiliations:** 1 Pharmacology, Jawaharlal Institute of Postgraduate Medical Education and Research, Puducherry, IND; 2 Preventive & Social Medicines, Jawaharlal Institute of Postgraduate Medical Education and Research, Puducherry, IND

**Keywords:** primary care, out of pocket expenditure, health for all, public health facilities, essential medicines

## Abstract

Introduction

The supply of essential medicines is one of the vital components of primary health care. One of the important objectives of Indian health policy is to provide all the essential medicines at an affordable cost for the public. The performance of healthcare facilities is directly affected by the supply of essential medicines. This study was conducted to check the availability of key essential medicines in selected public healthcare facilities of the South Indian Union Territory.

Methods

A snapshot survey was conducted between March 2019 and February 2020 in 10 selected public health facilities to assess the availability of 50 key essential medicines. Percentage availability for all surveyed medicines for the individual facility as well as percentage availability of individual medicines in all surveyed health facilities was calculated.

Results

Percentage availability of 50 key essential medicines in 10 surveyed public health facilities was found in a range of 66 to 80%. Out of 50 medicines, 26 (52%) medicines were available in more than 80% of health facilities while six (12%) medicines were available in less than 30% of surveyed facilities.

Conclusion

This study reported the high availability of essential medicines in public health facilities as compared to similar studies done in other parts of India but the availability of some essential medicines was found sub-optimal and needs to be improved.

## Introduction

Health is declared a fundamental human right [1]. As per World Health Organization (WHO), a health care system provides health care services at an affordable cost to the public [2]. One of the important components of the health care system is medicine. According to WHO, a major and considerable part of household expenses goes on medicines [3]. Access to medicines is one of the vital components of primary healthcare [1]. In 1977, WHO formed the concept of essential medicine [4,5]. One of the primary objectives of the essential drug concept was to maintain a regular supply of the required medicines at primary health centres [6]. Essential medicines are defined by WHO as “medicines that satisfy the health care need of the majority of the population”. Essential medicines are medicines to be available at all times in required amount, required dosage forms, with optimal quality and appropriate information, and at a price that individuals can afford [7]. A proper supply of safe, quality and affordable medicines to the public is the basis to achieve universal health coverage [8,9]. Access to medicines will be a pre-requisite for the implementation of “health rights” [6]. Target 8e of MDG (Millennium Development Goals) goals also stated that there is a need to improve the access of essential medicines, especially for low and low-middle income countries [3]. India consists of 33 federal states/union territories that are ruled by different political parties. In India, both the central as well as state governments play important role in health care management. India’s public health expenditure has remained between 1.2% and 1.6% of GDP between 2008-09 and 2019-20. In 2020-21, there is an increase of 3.9% in allocation over the revised estimates of 2019-20 [10].

One of the important objectives of Indian health policy is to provide all the essential medicines at an affordable cost for the public [11]. In developing countries like India, the availability of medicines is irregular, especially in public health facilities [12]. Surveys done in different parts of India showed a lack of availability of essential medicines, especially in public health facilities [13]. The performance of healthcare facilities is directly affected by the supply of essential medicines [4,12]. There is no study regarding the availability of essential medicines in Puducherry public health facilities. This survey was conducted to monitor the availability of 50 key essential medicines in Puducherry public health facilities. Data from this study could be beneficial to healthcare policymakers to know the present situation regarding essential medicines availability in Puducherry and suggest ways to improve the medicines availability that will bring benefit to patients.

## Materials and methods

Study design

A snapshot survey was designed to check the availability of 50 key medicines in 10 selected public health faculties of Puducherry.

Study site and duration

This study was conducted between March 2019 and February 2020. The study was carried out in Puducherry which is the largest district of Pondicherry union territory and consisted of an adequate number of health facilities functioning under both the state and central government.

Study population

A sample of 10 public health facilities was selected based on their direction. Public health facilities were selected based on simple random sampling. We selected a total of nine primary health centres and one tertiary care hospital. Out of the nine primary health centres, seven primary health centres are functioning under state government and two primary health centres are functioning under the central government.

Selection of medicines for survey

A checklist of key medicines to be surveyed was prepared as per WHO and HAI (Health Action International) guidelines [14]. The checklist contains a total of 50 key essential medicines which includes a Global and WHO-SEARO (South East Asia Region Origin) regional list of 30 medicines and a supplementary list of 20 medicines. The supplementary list was prepared by selecting 20 medicines from the National List of Essential Medicines (NLEM) of India 2015. A supplementary list was prepared by selecting those medicines which are being commonly used to treat common ailments at primary care levels and should be present at all times in primary health centres.

Data collection

One time survey was conducted in each selected health facility. Data collection was done by qualified and trained medical officials. The feasibility of data collection was checked by conducting a pilot study. Before visiting health facilities, a checklist of medicines to be surveyed was kept confidential to avoid bias. Data on the availability of 50 key essential medicines were collected on the day of the survey by direct observation of the pharmacy of individual health facilities in the presence of pharmacists and medical officers.

Data analysis

Data were entered in Microsoft excel (Microsoft® Corp., Redmond, WA) and analyzed for each public health facility separately. Percentage availability for all 50 surveyed medicines for the individual facility (Number of surveyed medicines available in health facility/total number of medicines surveyed) was calculated. The percentage availability of individual medicines in all surveyed health facilities (Number of health facilities in which individual medicine was available/total number of health facilities surveyed) was also calculated. Percent availability >80% is considered as high and <30% is considered as low. Data were analyzed by using Microsoft excel.

Ethical approval

This study was approved by the Institutional Ethical Committee of Jawaharlal Institute of Postgraduate Medical Education & Research, Puducherry (JIP/IEC/2018/338), India. Administration approval for collecting data from state government public health facilities was also taken from the Directorate of Health & Family Welfare, Government of Puducherry (585/DHFWS/PA/2019).

## Results

**Figure 1:**
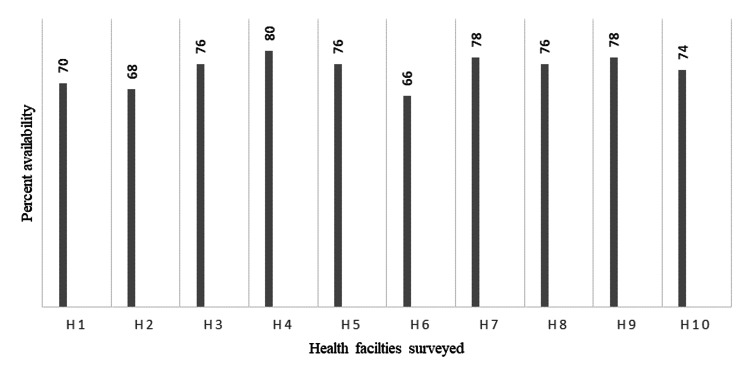
Percent availability of 50 key essential medicines in 10 public health facilities of Puducherry. H: health facility

The median availability of 50 key essential medicines in 10 surveyed public health facilities was 76%. The overall mean availability of 50 key essential medicines in state government primary health centres (PHCs), central government PHCs and tertiary care teaching hospital was 72.2% (range 66% to 80%), 77% (range 76% to 78%) and 74%, respectively. Percentage availability of surveyed essential medicines in 10 health facilities is given in Figure 1.

**Figure 2:**
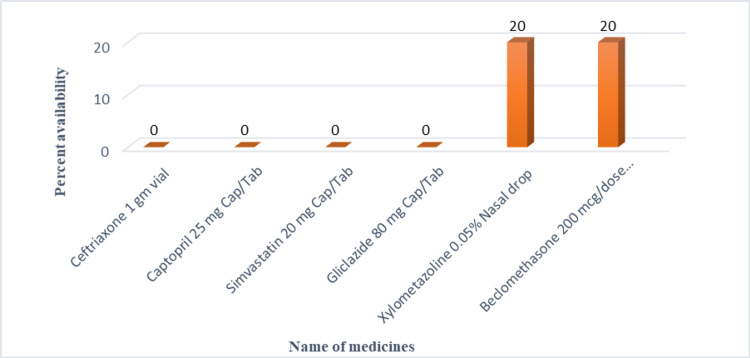
Medicines reported overall availability of less than 30 percent in public health facilities of Puducherry.

Out of 50 surveyed medicine 24 medicines in state government PHCs, 36 medicines in central government PHCs and 37 medicines in a tertiary care teaching hospital were found to be available at all the surveyed health facilities (100% availability). In state government PHCs, seven medicines (Captopril, Simvastatin, Gliclazide, Fluoxetine, Beclomethasone, Xylometazoline, and Ceftriaxone vial) were not available in any of surveyed health facilities (zero percentage availability) while four medicines (Amoxicillin dry syrup, Diethylcarbamazine, Enalapril, and Chloroquine) were available only in 42.85% of health facilities and one medicine (Hydrochlorothiazide) was available only in 14.28% of surveyed health facilities. In central government PHCs, eight medicines (Amoxicillin dry syrup, Ceftriaxone, Captopril, Simvastatin, Ranitidine, Gliclazide, Gentamicin eye drop, and Chloroquine) were found to have zero percentage availability. In a tertiary care teaching hospital, 13 medicines (Ibuprofen, Amoxicillin dry syrup, Ceftriaxone, Cotrimoxazole suspension, Captopril, Simvastatin, Ranitidine, Gliclazide, Chloroquine, Xylometazoline, Antacid, Multivitamins and Zinc supplement) were not available. Medicines with overall percentage availability of less than 30 in surveyed health facilities are shown in Figure 2.

A complete list of percentage availability of individual medicine in surveyed health facilities is given in Table 1.

**Table 1:** Percent availability of individual medicine surveyed in public health facilities of Puducherry, India n = number of health facilities surveyed; PHC = Primary health centre; SEARO = South East Asia Region Origin.

S. No	Name of medicine	Dosage form	Strength	Percent availability			
				State govt. PHCs (n = 7)	Central govt. PHCs (n = 2)	Tertiary care teaching hospital (n = 1)	Overall mean availability (n = 10)
GLOBAL and WHO-SEARO Regional List of 30 Medicines							
1	Diclofenac	Cap/Tab	50 mg	100%	100%	100%	100%
2	Ibuprofen	Cap/Tab	400 mg	85.71%	100%	0%	80%
3	Paracetamol	Suspension	24 mg/ml	100%	100%	100%	100%
4	Amoxicillin	Cap/Tab	500 mg	100%	100%	100%	100%
5	Amoxicillin	Dry syrup	250 mg/5ml	42.85%	0%	0%	30%
6	Ceftriaxone	Vial	1 gm	0%	0%	0%	0%
7	Ciprofloxacin	Cap/Tab	500 mg	100%	100	100%	100%
8	Co-trimoxazole (sulfamethoxazole + trimethoprim)	Suspension	200 mg + 40 mg/5 ml	100%	50%	0%	80%
9	Doxycycline	Cap/Tab	100 mg	71.42%	100%	100%	80%
10	Diethylcarbamazine	Cap/Tab	50 mg	42.85%	100%	100%	60%
11	Metronidazole	Cap/Tab	400 mg	100%	100%	100%	100%
12	Amlodipine	Cap/Tab	5 mg	100%	100%	100%	100%
13	Atenolol	Cap/Tab	50 mg	85.71%	50%	100%	80%
14	Enalapril	Cap/Tab	5 mg	42.85%	100%	100%	60%
15	Captopril	Cap/Tab	25 mg	0%	0%	0%	0%
16	Atorvastatin	Cap/Tab	10 mg	85.71%	100%	100%	90%
17	Simvastatin	Cap/Tab	20 mg	0%	0%	0%	0%
18	Ranitidine	Cap/Tab	150 mg	100%	0%	0%	70%
19	Omeprazole	Cap/Tab	20 mg	100%	100%	100%	100%
20	Metformin	Cap/Tab	500 mg	100%	100%	100%	100%
21	Glibenclamide	Cap/Tab	5 mg	100%	100%	100%	100%
22	Gliclazide	Cap/Tab	80 mg	0%	0%	0%	0%
23	Clotrimazole	Cream	1%	100%	100%	100%	100%
24	Gentamicin	Eye drop	0.3%	57.14%	0%	100%	50%
25	Amitriptyline	Cap/Tab	25 mg	57.14%	100%	100%	70%
26	Diazepam	Cap/Tab	5 mg	71.42%	100%	100%	80%
27	Fluoxetine	Cap/Tab	20 mg	0%	100%	100%	30%
28	Phenytoin	Cap/Tab	100 mg	85.71	100%	100%	90%
29	Beclomethasone	Inhaler	200 mcg/dose	0%	50%	100%	20%
30	Salbutamol	Inhaler	100 mcg/dose	57.14%	50%	100%	60%
Supplementary list of 20 medicines							
31	Lignocaine	Vial	2%	100%	100%	100%	100%
32	Paracetamol	Tab	500 mg	100%	100%	100%	100%
33	Chlorpheniramine	Tab	4 mg	100%	100%	100%	100%
34	Mebendazole	Tab	100 mg	100%	100%	100%	100%
35	Co-trimoxazole (sulfamethoxazole + trimethoprim)	Tab	400 mg + 80 mg	100%	100%	100%	100%
36	Cough syrup	Syrup	-	57.14%	50%	100%	60%
37	Rifampicin, Pyrazinamide, Isoniazid	Tab/Cap	120 + 300 + 50 mg	85.71%	50%	100%	80%
38	Chloroquine	Tab	150 mg	42.85%	0%	0%	30%
39	Ferrous salt	Tab	60 mg	100%	100%	100%	100%
40	Folic acid	Tab	1.5 mg	100%	100%	100%	100%
41	Silver sulfadiazine	cream	1%	71.42%	100%	100%	80%
42	Hydrochlorothiazide	Tab	25 mg	14.28%	100%	100%	40%
43	Xylometazoline	Nasal drop	0.05%	0%	100%	0%	20%
44	Antacid	Syrup	-	100%	100%	0%	90%
45	Metoclopramide	Tab	10 mg	100%	100%	100%	100%
46	Dicyclomine	Tab	10 mg	85.71%	100%	100%	90%
47	Oral rehydration salt	Powder	-	100%	100%	100%	100%
48	Zinc supplement	Tab	20 mg	71.42%	100%	0%	90%
49	Calcium salt	Tab	500 mg	100%	100%	100%	100%
50	Multivitamins	Tab	-	100%	100%	0%	90%

## Discussion

The concept of essential medicines was developed to promote rational use, lower cost, and improve access. The rational use of medicines (RUM) is defined as “Patients receive medications appropriate to their clinical needs, in doses that meet their requirements, for an adequate period, and at the lowest cost to them and their community” [15]. Availability of essential medicines plays a major role in rational prescribing by primary care physicians. WHO developed core drug use indicators (five prescribing indicators, five patient care indicators, and two health facility indicators) to measure rational drug use at the primary health level. Out of five prescribing indicators, “percentage availability of essential medicines” is one of the core prescribing indicators [16]. Non-availability of essential medicines at the primary care level leads to prescribing of other alternatives which could further increase the cost of therapy and out-of-pocket expenditure but also promote irrational prescribing practices. Various studies conducted in public healthcare sectors in developing and transitional countries provide evidence that successful implementation of essential medicines policies leads to rational and quality use of medicines [17]. This survey has provided a snapshot of the availability of 50 key essential medicines in public health facilities of Puducherry. WHO/HAI has set criteria of 80% for medicine availability as high [18,19] against which we reported in our survey that overall median percentage availability in 10 selected public health facilities was 76% which was slightly lower than the benchmark set up by WHO and HAI. Poor medicines availability in the government sector could result from various factors such as low budget allocation for medicines, purchasing non-essential medicines, inability to forecast needs accurately, and inefficient drug supply chain management [20,21]. Our findings are higher than reported in similar studies conducted in Delhi, Odisha, and five Indian states where availability of surveyed essential medicines in the public sector was found to be 41.3% in Delhi [22], 17% in Odisha [23] and 0 to 30% in five Indian states [24], respectively. Our value is higher than the study conducted in two states (Punjab and Haryana) where the overall availability of medicines was 45.2% in Punjab and 51.1% in Haryana [25]. Our findings are better than those found in a previous study conducted in Shaanxi Provinces of China that has shown that the mean availability of originator brands and generic medicines in the public sector was 7.1% and 20.0%, respectively [26]. Malaria is a commonly occurring disease and contributes to significant morbidity and mortality of children. In our study, we found that antimalarial drug chloroquine was available only in three health facilities (30% availability) which was lower than found in a similar study conducted in Odisha (42.7%) [23]. Zinc supplement is essential medicine for children and is also listed in the National Rural Health Mission list of essential medicines. In our study, we found the availability of zinc sulfate was 70% which is higher than the value (<50%) reported in a similar study conducted in 129 health centres of different states of India [27]. In the current study we found that the anti-diabetic drug Glibenclamide was available in all surveyed health facilities (100% availability) which is similar to the value found in Karnataka (100%) but higher than that found in Chennai (95%), Haryana (83.3%), Maharashtra (15%) and in West Bengal (3.8%) [24]. Diarrhoea is a frequently reported illness at primary health care centres and affects both adults as well as children. Oral rehydration salt (ORS) is essential medicine to treat diarrhoea and is listed in both WHO and Indian list of essential medicines. In our study, we found that ORS was available at all the surveyed health facilities (100% availability). A similar value was found in Karnataka, Tamil Nadu, West Bengal, Kerala, Orissa, Maharashtra, Gujarat, Andhra Pradesh, Meghalaya, Mizoram, and Chhattisgarh whereas low availability of ORS was reported in four states, i.e., Jammu and Kashmir (53.3%), Punjab (86.7%), Nagaland (75%) and Madhya Pradesh (0%) [27]. Non-availability of essential medicines can also lead to nonadherence. A study conducted in Delhi, India reported that one of the determinants of non-adherence to anti-hypertensive treatment among patients attending PHCs was running out of drug stocks [28] which has also been observed in other studies [29,30].

Drug shortages have been described as “public health crises” due to their threats to the prevention of acute and chronic diseases. The advantages of greater access to generic medicines in public health facilities include the benefit to patients in receiving all the care needed at a single point. Results of this survey indicate that most of the medicines were available at all the surveyed health facilities. In our study, we found that few medicines were not available in all the surveyed health facilities and their availability needs to be improved. Availability of essential medicines in Puducherry can be improved by allocating more funds for medicines, regular monitoring of medicines stock, purchasing medicines based on essential medicine concepts and strengthening of drug supply chain system [31,32]. Our study has certain limitations such as availability is determined for the selected list of survey medicines and, therefore, does not account for the availability of alternate strengths or dosage forms, or therapeutic alternatives. We did not assess what were the reasons for non-availability. Was it a temporary stock out or prolonged stock out situation? What was the rate of utilization or demand for these drugs at the PHCs - sometimes drugs expire because patients are not available for the condition for which the drug is needed. In that case, even an otherwise essential drug may not be placed on drug demand by the medical officer for efficient inventory control. So a public health concern would truly exist only if an essential drug is unavailable despite its demand placement by the medical officer(s) at a primary care facility.

## Conclusions

This was a snapshot survey conducted to check the availability of 50 key essential medicines in public health facilities of Puducherry. More than 60% of medicines were available in all surveyed health facilities. The overall mean availability in state government PHCs was found to be lower than central government PHCs and tertiary care teaching hospitals. The majority of the low-income population relies on public health care facilities. Puducherry needs to implement essential drug concepts by preparing state essential medicines list (EML) and mandate that physicians in primary healthcare facilities prescribe generic medicines and follow EML. The EML can help the state to rationalize the purchasing and distribution of medicines, thereby reducing costs to the health care system as well as the patient. The ministry of chemical and fertilizers (department of pharmaceuticals) started Pradhan Mantri Jan Aushadhi Yojna Scheme with the aim of providing quality generic medicines at cheap price through Jan Aushadhi Centers to reduce out-of-pocket expenses of patients. Puducherry state government should provide space in PHCs or any other suitable locations for running of the Jan Aushadhi Stores. This will help patients buy medicines at cheaper cost which are not available at PHCs. Medicines’ availability in Puducherry can be improved by allocating more funds for medicines, regular monitoring of medicines stock, purchasing medicines based on essential medicine concepts and strengthening of drug supply chain system.
